# On the Use of Corrosive Sublimate in Dysentery

**Published:** 1841-08-21

**Authors:** William S. Helmuth


### MEDICAL EXAMINER.

DEVOTED TO MEDICINE, SURGERY, AND THE COLLATERAL SCIENCES.

No. 34.] PHILADELPHIA, SATURDAY, AUGUST 21, 1841. [Vol. IV.

### ORIGINAL COMMUNICATION.

*On the use of Corrosive Sublimate in Dysentery.*

By William S. Helmuth, M. D.

To the Editors of the Medical Examiner.

*Gentlemen,*—My present object is merely to invite the attention of the profession, through the medium of your pages, to a remedy which in my hands has proved promptly efficacious in the treatment of severe cases of dysentery. The prevalence of this disease at this season affords physicians frequent opportunities of testing the correctness of my experience, and my desire to have it confirmed furnishes a reason for this brief and hasty communication.

I shall therefore forbear detailed histories of the cases, and only remark that they presented the usual characteristics of the disease, and, in some instances, in an aggravated form, the discharges being very frequent, consisting of blood and mucus, with much griping, tenesmus, and abdominal pain.

The medicine used was the corrosive chloride of mercury, and in the following manner, viz. :

℞. Hydrar. corros. chlorid. gr. ss.

Aquæ fluvialis ℥ss. M. fiat.

*Dose.*—Three drops every three hours in a tea-spoonful of cool water.

This dose was repeated until the symptoms began to moderate, from which period it was exhibited less frequently. A favourable change sometimes occurred before the expiration of the first three hours, and always after the administration of the second dose. A strict antiphlogistic regimen was enjoined.

As soon as much relief was experienced, the remedy was altogether discontinued.

The employment of so novel a remedy in the treatment of this often vexatious and dangerous disease, was suggested from its known physiological action upon the system ; and it remains for future experiments to determine whether the same physiological rule must also guide us in the choice of all other remedial agents.

